# Bactericidal and Antiviral Bionic Metalized Nanocoatings

**DOI:** 10.3390/nano12111868

**Published:** 2022-05-30

**Authors:** Mikhail Kryuchkov, Jozef Adamcik, Vladimir L. Katanaev

**Affiliations:** 1 Department of Cell Physiology and Metabolism, Translational Research Centre in Oncohaematology, Faculty of Medicine, University of Geneva, 1211 Geneva, Switzerland; mikhail.kryuchkov@unige.ch; 2 National Center of Competence in Research Bio-Inspired Materials, Adolphe Merkle Institute, University of Fribourg, 1700 Fribourg, Switzerland; jozef.adamcik@unifr.ch; 3 Institute of Life Sciences and Biomedicine, Far Eastern Federal University, 690922 Vladivostok, Russia

**Keywords:** nanocoatings, bionic, self-assembly, bactericidal, antiviral, *Drosophila*, *Anopheles*, Retinin, Turing

## Abstract

In diverse living organisms, bionanocoatings provide multiple functionalities, to the surfaces they cover. We have, previously, identified the molecular mechanisms of Turing-based self-assembly of insect corneal nanocoatings and developed forward-engineering approaches to construct multifunctional soft bionic nanocoatings, encompassing the *Drosophila* protein Retinin. Here, we expand the versatility of the bionic nanocoatings, by identifying and using diverse Retinin-like proteins and different methods of their metallization, using nickel, silver, and copper ions. Comparative assessment, of the resulting bactericidal, antiviral, and cytotoxic properties, identifies the best protocols, to construct safe and anti-infective metalized bionic nanocoatings. Upscaled application of these protocols, to various public surfaces, may represent a safe and economic approach to limit hazardous infections.

## 1. Introduction

**Figure 1:**
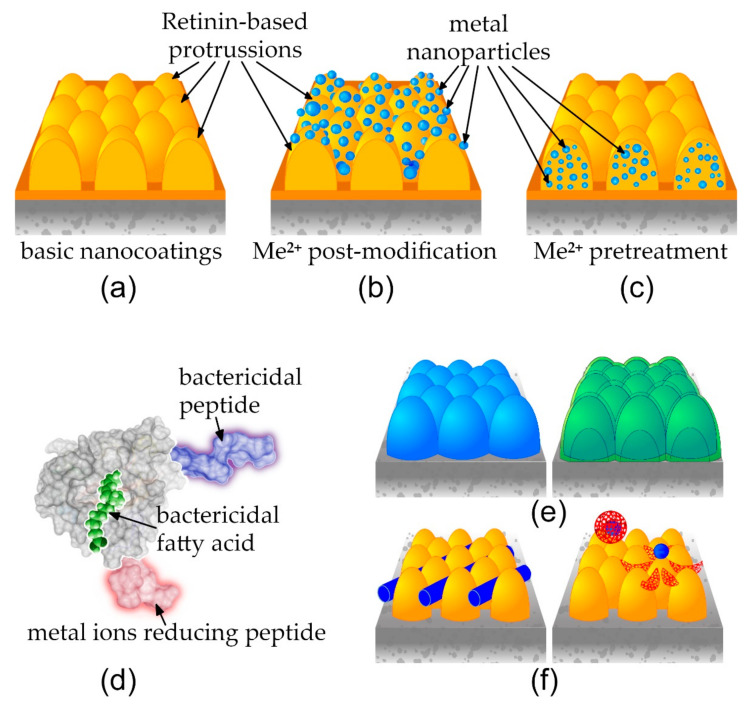
Functionalization of bionic nanocoatings. Retinin-based nanocoatings (**a**) can be metalized, by coalescence of metal nanoparticles after the nanocoating formation (**b**) or by bioconjugation before the process of nanocoating self-assembly (**c**). Possible functionalizations include modifications of the core protein and fatty acid components, to build nanocoatings with enhanced anti-infective properties (**d**). Further, single- or multi-layering with metals or other compounds can be envisioned (**e**), along with integration of various nanoscale objects, such as carbon nanotubes or complex multi-compound nanoparticles (**f**), for diverse applications from medicine to electronics.

Representatives of different domains of life have evolved diverse nanocoatings, rendering liquid-handling, photonic, adhesion, bactericidal, and other functionalities to the surfaces they cover [1,2,3,4,5]. Understanding of the molecular and physical mechanisms, which operate at formation of the bionanocoatings, is necessary for eventual biomimetic developments, for technological applications. Biological patterning at the micro- and macro-scale has, in many cases, been shown to follow Alan Turing’s reaction–diffusion pattern-formation mechanism [6,7]. We have, previously, shown that in diverse arthropods’ surfaces, such as eye corneae of butterflies, tarsal segments of stick and leaf insects, or light-emitting abdomena of fireflies, bionanocoatings rendering anti-reflective, light polarization, anti-wetting, or adhesion properties follow the same principles of Turing patterning [3,8,9,10,11,12,13,14,15]. The reaction–diffusion mechanism is based on interactions of two morphogens: a slow-diffusing activator and a fast-diffusing inhibitor [6,7]. Through forward and reverse engineering in *Drosophila*, we have shown that the corneal protein Retinin acts as the Turing activator morphogen and as corneal waxes—the inhibitor in formation of anti-reflective and anti-wetting nanocoatings, covering the insect’s eye surfaces [11]. Recombinant production of Retinin or Retinin-like proteins from *Drosophila* permits reconstruction of bionic nanocoatings, upon admixtures with waxes on artificial surfaces, providing them with diverse liquid-handling and light-operating properties [11,12]. We have, further, proposed that by using our bio-inspired creation of soft nanocoatings, as the basis for composite nanomaterials, multiple further functionalities could be added, e.g., for electronics or anti-infective applications [11,12] (Figure 1). In the current work, we focus on two approaches for the metallic armament of Retinin or Retinin-like protein- and wax-based bionic nanocoatings gaining bactericidal and antiviral properties.

Each year, antibiotic-resistant bacteria kill over 1.2 million people [16]. According to the World Health Organization, drug resistance of microbes could cause 10 million deaths per year by 2050, damaging society and the economy more than the COVID-19 pandemic [17]. Speaking of the latter, viral infections represent another major threat to public health, swapping through humankind at a speed often exceeding the time needed to raise vaccines against them [18].

To protect from infections, a barrier decreasing the spread of infectious agents among people should be created. Various surfaces in public spaces—e.g., hospital beds, seats on buses, or even children’s playgrounds [19]—represent hazardous reservoirs for bacterial and viral infections. For example, COVID-19 virus particles can survive on plastic and stainless steel, for up to three days [20], whereas bacteria, such as *E. coli* and *S. aureus*, can survive for several months [21]. One can, thus, aim at functional modifications of public surfaces, in order to convert them from pathogen reservoirs to an anti-infective barrier. Deposition of silver and copper nanoparticles can be considered as one approach to this task, as bacteria and viruses have not developed resistance to these metals over thousands of years, in sharp contrast to antibiotic resistance [22,23,24,25]. Pathogen elimination, by naturally structured surfaces such as bactericidal coatings of cicada wings [26], has also been successful in fighting bacteria for millions of years, without apparent bacterial-resistance mechanisms emerging. Nowadays, developments of anti-infective nanocoatings include application of Ag, Zn, and Cu ions or nanoparticles; incorporation of photosensitizers, such as TiO_2_ or rose bengal; coating by polyelectrolytes and peptides; incorporation of antibiotics or graphene; micro-nano-structuring of metals or polymers. However, these approaches have clear limitations related to the issues, such as cytotoxicity, complexity of application, or insufficient efficiency [27,28,29,30,31,32,33].

We here describe antibacterial and antiviral effectiveness of reverse-engineered bionic nanocoatings functionalized with metals. The protein (Retinin or Retinin-likes from *Drosophila* or other insects) at the basis in such nanocoatings makes them competent to react with metals’ ions, reducing them from salts, such as AgNO_3_ or CuCl_2_, to form metallic nanoparticles. The amino acids of proteins, Trp, Tyr, Ser, and Thr, have been shown to bind metal ions, leading to their reduction, coalescence, and, ultimately, nucleation in metal nanoparticles [34,35,36]. We show that functionalization of protein-based bionic nanocoatings, with metal nanoparticles, achieves increased anti-infective capacities, compared to pure metallic coatings. Moreover, the methods we propose could, additionally, resolve the problem of the toxicity of pure nanoparticles, as transformation of the metal nanoparticles into protein-based bioconjugates reduces the cellular uptake and the resultant toxicity of metals [37].

## 2. Materials and Methods

### 2.1. Preparation of Eye Samples of Anopheles gambiae for Atomic Force Microscopy (AFM) and Proteomic Analyses

Eyes of adult mosquitoes *Anopheles gambiae* were cut off the head capsules, with a sharp needle. Retinal material was isolated, by washing the eyes with a pipette tip; the resulting suspension was dried. Corneal material was, additionally, washed three times in water and processed for AFM. Alternatively, the corneal (and control retinal) samples collected from 15 insects were subjected to 15% polyacrylamide gel-electrophoresis in SDS (SDS-PAGE), after 15 min of boiling in the sample buffer (62.5 mM Tris-HCl, pH 6.8; 10% glycerol; 2% SDS; 1% β-mercaptoethanol; a trace of bromophenol blue).

### 2.2. AFM

Topography data were collected in the tapping mode, using an XE-100 (Park Systems) microscope and PPP-NCHR cantilevers (Nanosensors, radius of curvature < 25 nm), at a micro-nanotechnology facility (Haute École Specialisée de Suisse Occidentale, Geneva, Switzerland). Young’s modulus measurements were performed, using an NX10 (Park Systems) microscope and Tap300AI-G (Budget Sensors) cantilevers, with the nominal spring constant k = 40 N/m and curvature radius 10 nm. Measurements were done in the PinPoint^TM^ mode, with the following parameters: speed 30 µm/s and set point 290 nN. Quantification of the elastic modulus in this mode was based on the Hertzian model. All scans were, initially, measured as 4.5 × 4.5 μm squares, with the resolution of 256 × 256. Gwyddion software version 2.55 [38] was used for visualization and quantitative analysis.

### 2.3. Mass-Spectrometry

Following SDS-PAGE, protein bands visible after gel staining were cut off. In-gel trypsin digestion and mass-spectrometry was performed at the Protein Analysis Facility of the University of Lausanne.

### 2.4. Cloning of Genes for Retinin and Retinin-Like Proteins

Following the cloning for recombinant expression of *Drosophila melanogaster* Retinin [11] and Retinin-like CG13059 [12], DNAs encoding the Retinin-like proteins of *D. melanogaster* CG42718 and Nplp3 were amplified from the cDNAs (catalog ## FI16837 and RH16169, respectively, obtained from the *Drosophila* Genomics Resource Center) using the following oligonucleotides: CG42718 forward (ctgtatacatatgagaggatctcaccatcaccatcaccattgccaaatccatcataatct), CG42718 reverse (ctgttgactcgagccttaatgtgggcagatcccttgtca), Nplp3 forward (ctgtatacatatgagaggatctcaccatcaccatcaccatgccccagctcccgctcctgc), and Nplp3 reverse (ctgttgactcgagcctcaaccgatcacaactggtccca). DNA encoding of the Retinin-like protein CPR10 of *Anopheles gambiae* was amplified from cDNA made by reverse transcription (PrimeScript RT Reagent Kit, Takara) from pupal samples (12 h after pupariation), using oligonucleotides: CPR10forward (ctgtatacatatgagaggatctcaccatcaccatcaccatcaacagtatggccagcagct) and CPR10reverse (agcgcaggcggccgcttttagttgcgcagcagaacgg). All the forward oligonucleotides encompass an RGSHis-tag coding sequence, adding the tag to the N-termini of proteins, upon recombinant production. The PCR products were subcloned into the NdeI and XhoI sites of pET23b.

### 2.5. Purification of Retinin and Retinin-Like Proteins

We used the Rosetta-gami (Novogen, Sacramento, CA, USA) *Escherichia coli* strain for recombinant protein expression, after IPTG induction (incubation at 37 °C until OD_600_ = 0.6, final concentration of IPTG: 0.2 mM, post-induction growth at 19 °C: 12 h). The bacterial mass was lysed by a French press (Constant Systems, Daventry, UK). RGSHis-tagged proteins were purified, using HisPur Ni-NTA resin (ThermoFisher Scientific, Waltham, MA, USA) and a Superdex 200 Increase 10/300 GL column (Cytiva, Washington, DC, USA), as described [11,12].

### 2.6. Fatty Acid Emulsion Preparation

In total, 5 mg of decanoic acid (Sigma-Aldrich, Burlington, VT, USA) was added to tubes with 10 mL 0.5% SDS solutions in TBS (150 mM NaCl, 50 mM Tris-HCl, pH 7.6) and sonicated in a water bath (AL 04-04, Advantage-Lab), for 2 h at 80 °C.

### 2.7. Surface Treatment

#### 2.7.1. Basic Protein-Based Nanocoatings

The mixture of Retinin or Retinin-like proteins (0.7 mg/mL in TBS) and a decanoic acid emulsion were applied to the surface of a polystyrene petri dish (Sigma-Aldrich, Burlington, VT, USA), at the protein, with a fatty acid solutions volume ratio of 4:1. Next, this solution was dried at room temperature and rinsed 3× with Milli-Q water.

#### 2.7.2. Post-Assembly Metallization

The coated surfaces were covered with 100 mM NiSO_4_, AgNO_3_, or CuCl_2_, for 1 h; next, the liquid was removed, and the surface was rinsed 3× with Milli-Q water.

#### 2.7.3. Pre-Assembly Metallization

Retinin or Retinin-like proteins (0.7 mg/mL in TBS) were incubated with a metal salt solution (final concentration 10 μM), for 4 h. Next, the mixture of the protein and the decanoic acid emulsion was applied to the surface, at the ratio of 4:1 (volume:volume). This solution was dried at room temperature and rinsed 3× with Milli-Q water. A mixture of BSA (bovine serum albumin, Roche, Basel, Switzerland) with AgNO_3_ was used as a control, following the same protocol. Additionally, negative controls with the metal ions absorbed by plastic were prepared, by applying 10 μM AgNO_3_ or CuCl_2_ to the plastic, with drying and washing as above.

### 2.8. SEM and EDS Analyzes

A scanning electron microscope (SEM, TESCAN Mira 3 LM Field Emission, Brno-Kohoutovice, Czech Republic) was used to image the sample, which was sputter coated with a 2 nm thick layer of gold, using a 208 HR sputter coater (Cressington Scientific Instruments, Watford, UK). The images were taken with an SEM, operated at an acceleration voltage of 5 kV. The elemental composition of sample was studied, using energy-dispersive X-ray (EDX) mapping analysis, using an EDX detector (EDAX Ametek Materials Analysis Division, Octane Pro, Mahwah, NJ, USA) integrated into the SEM.

### 2.9. Anti-Infective Activity Measurement

The metal-conjugated nanocoatings’s antibacterial and antiviral activities were measured, according to the ISO 22196:2011 protocols (iso.org/standard/54431.html, accessed 30 May 2022), with minor changes, as detailed below.

#### 2.9.1. Bactericidal Activity Measurement

In total, 10 μL bacterial suspension (Top 10f *E. coli* strain, Thermo Fisher) at 10,000 CFU/mL in LB medium was applied to the surfaces, prepared as described above, covered by a glass coverslip, and cultured for 6 h at 37 °C. Next, the liquid was collected, and the surfaces were washed with additional 100 μL LB medium, thoroughly scraping the surface with a plastic tip. The two resultant bacterial suspensions were united and applied to solid LB agar medium in 3.5 cm Petri dishes. The numbers of colonies grown after 16 h at 37 °C were manually counted.

#### 2.9.2. Antiviral Activity Measurement

In total, 10 μL suspension of lentiviral particles produced from HEK-293 cells after transfection of pPAX2, PVSVG and pSD28-GFP [39] was applied to the surfaces, prepared as described above, covered by a glass coverslip, and incubated for 6 h at 37 °C. Next, the liquid was collected, and the surfaces were washed with an additional 100 μL of DMEM (Thermo Fisher, Waltham, MA, USA), thoroughly scraping the surface with a plastic tip. The two resultant viroid suspensions were united and precipitated in PEG-8000 PBS solution [40]. DMEM, with Polybrene 10 µg/mL (Sigma), containing concentrated lentivirus particles, was added to HEK-293 cells, in transparent 96-well plates (Thermo Fisher, 10,000 cells/well). The lentivirus-induced GFP fluorescence was measured, after 48 h of incubation at 37 °C, 5% CO_2_, by an Infinite M Plex multifunctional plate reader (Tecan).

### 2.10. Cytotoxicity Test

Following ISO 10993-5:2009 (iso.org/standard/36406.html, accessed 30 May 2022), we assessed the cytotoxicity of various coatings with an MTT assay. DMEM (10 μL) was applied to the surfaces, prepared as described above, covered by a glass coverslip, and cultured for 6 h at 37 °C. Next, the liquid was collected, and the surface was washed with an additional 40 μL of DMEM, thoroughly scraping the surface with a plastic tip. The two liquids were united and added to HEK-293 cells in transparent 96-well plates (6000 cells/well). After 48 h incubation at 37 °C, 5% CO_2_, the medium was removed from the wells, and 20 µL of MTT reagent (triazolyltetrazoliumumbromide, Sigma), at 0.5 mg/mL in PBS, was added and incubated for 3.5 h at 37 °C. The solution was removed from wells, using a plate washer. Subsequently, 50 µL of DMSO was added and incubated for 5 min, and the optical density was measured by an Infinite M Plex multifunctional plate reader.

### 2.11. Statistical Analysis

Statistical processing (two-tailed *t*-test) of the data and graph plotting was performed with Prism 8 software (Dotmatics, Boston, MA, USA).

## 3. Results

### 3.1. Diversification of the Basic Retinin-Based Nanocoatings

**Figure 2:**
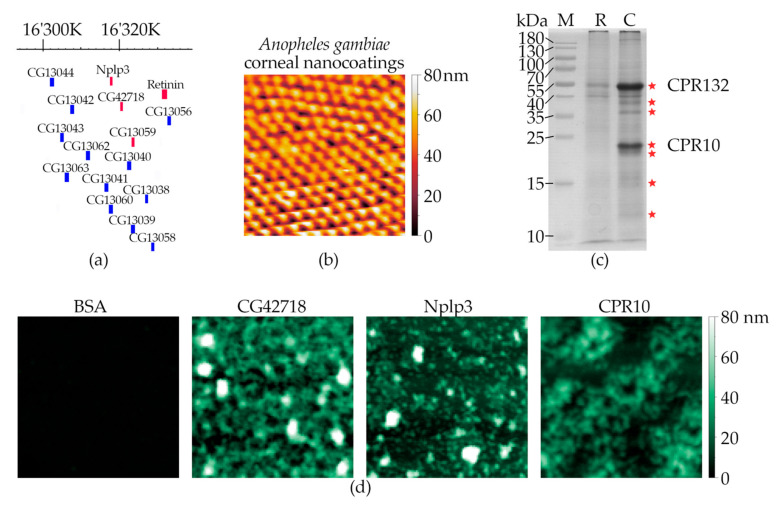
Identification of functional Retinin-like proteins. (**a**) On the chromosome arm 3 L of *D. melanogaster*, Retinin clusters together with several Retinin-like genes, encoding small-sized proteins with signal peptides, for extracellular secretion. Nplp3, CG13059, and CG42718 (marked in red, together with Retinin) were cloned and used in this work. The image is based on the GBrowse function of Flybase. Numbers on top are the chromosome’s nucleotide numbers. (**b**) AFM scan of *A. gambiae* corneal nanocoatings. (**c**) SDS-PAGE of retinal (R) and corneal (C) material from eyes of *A. gambiae*. Proteomic analysis of the corneal material identified two major proteins: CPR10 and CPR132. See Supplementary Materials Table S1, for peptides identified in the protein bands, marked with stars. (**d**) Topography of Retinin-like-based nanocoatings showed the self-assembly activity of these proteins, unlike of the control protein (BSA) but similar to that of Retinin and CG13059 [11,12]. Scans in (**b**,**d**) are 2 × 2 μm; surface height is indicated by the color scale, shown to the right of the images.

The gene encoding Retinin in *D. melanogaster* is located in a dense cluster of ca. 30 Retinin-like genes [12] (Figure 2a). We have, recently, shown that one of such Retinin-like genes, CG13059, can, also, produce functional bionic nanocoatings, upon admixtures with waxes on artificial surfaces [12]. Retinin is 191aa-long, while CG13059 is 155aa-long (20 kDa and 15.8 kDa, respectively). As Turing patterns, directly, depend on the diffusion properties of the morphogens [6,7] that are, in turn, dependent on the protein size [8,11], we selected, for further analysis, the two shortest proteins from the Retinin cluster: Nplp3 (CG13061) and CG42718 (8.7 kDa and 8.8 kDa, respectively).

Additionally, we identified CPR10 as the Retinin-like protein from the *Anopheles gambiae* corneae (Figure 2b), by methods similar to those we had previously applied to the *Drosophila* species [11] (Figure 2c, Supplementary Materials Table S1 and Figure S1; also, see Materials and Methods). To prove that the chosen proteins are, functionally, similar to Retinin, we investigated their ability to form nanostructured coatings, upon interaction with waxes. The tested Retinin-like proteins showed the ability to create nanostructured coatings with different heights, densities, and shapes (Figure 2d), similarly to Retinin and CG13059 [11,12]. We, thus, expanded the arsenal of Retinin-like proteins capable of forming bionic nanocoatings, and, next, moved to metalization of the nanocoatings.

### 3.2. Coalescence of Metal Nanoparticles on Top of Protein-Based Nanocoatings

We investigated two approaches to achieve the addition of metal nanoparticles to bionic nanocoatings, for anti-infective applications (Figure 1a–c). The first aimed at modification of pre-formed soft nanocoatings, by metal nanoparticles (coalescence of metal nanoparticles, Figure 1b). The second aimedat permitting metallization of the bionic nanocoatings, before their self-assembly (Figure 1c).

**Figure 3:**
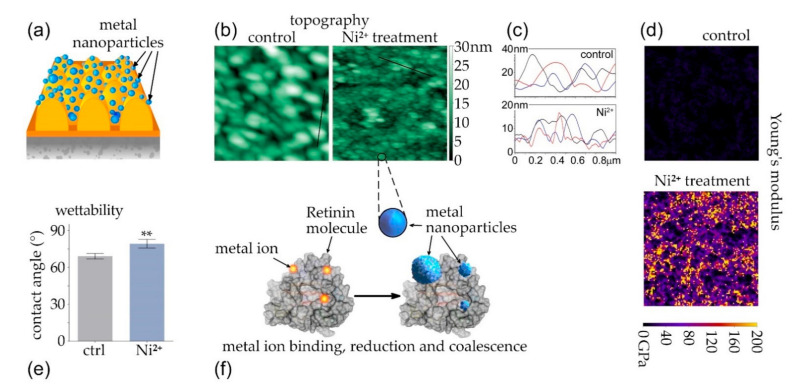
Armament with metal nanoparticles after nanocoating formation (**a**). Retinin-based bionic nanocoatings before (left, (**b**)) and after NiSO_4_ treatment (right, (**b**)). Black lines are the cross sections used to plot the heights. (**c**) Cross sections through the control (upper panel) and NiSO_4_-treated (lower panel) nanocoatings, showing representative heights of the nanostructures (three different cross sections are shown in different colors: black are from the images in (**b**), red and blue from other images). Measurement of the Young’s modulus of the same nanocoatings (**d**) and improved anti-wettability (**e**) confirms successful metallization, which is hypothesized to occur through metal ion binding, reduction, and metal-nanoparticle coalescence on the protein (**f**). Scans in (**b**,**d**) are 2 × 2 μm; surface height and Young’s modulus are indicated by the color scales, next to the images. Data in (**e**) are presented as mean ± SD, *n* = 4; ** indicates the *p*-value ≤ 0.01.

To test the effectiveness of the coalescence approach, we modified pre-formed Retinin-based nanocoatings, by incubating them with a NiSO_4_ solution (Figure 3a). AFM-based comparison of the topography of the nanocoatings, before and after NiSO_4_ incubation, suggests the appearance of metal nanoparticles, with a characteristic size from 20 nm to 100 nm (Figure 3b,c). To further prove metallization of the Retinin-based nanocoatings, we applied Young’s modulus measurements, which describe material elasticity, expecting a significant augmentation of this parameter, after metal armament of the protein-based nanocoatings. Indeed, the Young’s modulus of non-modified nanocoatings attained 10 GPa agreeing with the literature data on protein-covered surfaces [41], while that of the modified surface exceeded 200 GPa, agreeing with the data for nickel-covered surfaces [42] (Figure 3d).

We, also, studied wettability of the resultant metalized nanocoatings, finding that the contact angle of water droplets on the surfaces modified by Ni^2+^ significantly increased (Figure 3e), reflecting a reduction in the initial hydrophilicity of the Retinin-based nanocoatings [11].

We, thus, conclude that pre-formed Retinin-based nanocoatings can be, efficiently, metalized through the metal-ion binding, reduction, and coalescence of nickel nanoparticles (Figure 3f). Similarly, we produced silver- and copper-based armaments of the Retinin-based nanocoatings (see below).

### 3.3. Metal-Conjugated Retinins Retain the Capacity of Self-Assembly

**Figure 4:**
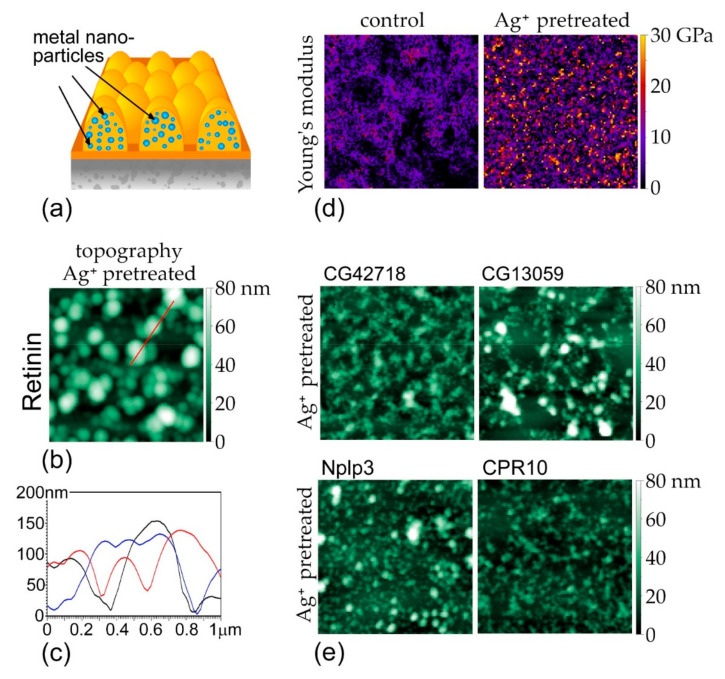
Armament with metal nanoparticles growing before the process of self-assembly (**a**) results in a pronounced topology of Retinin-based nanocoatings, incorporating silver nanoparticles (**b**). Red line is a cross section used to plot the heights. (**c**) Cross sections through Ag^+^-pretreated nanocoatings showing representative heights of the nanostructures (three different cross sections are shown in different colors, red is from the image in (**b**), black and blue from other images). (**d**) Young’s modulus of Ag^+^-pretreated nanocoatings is intermediate, between that of protein-based and metal-based surfaces. (**e**) Topology of silver-incorporating nanocoatings, based on Retinin-like proteins CG42728, CG13059, Nplp3, and CPR10. Scans in (**b**,**d**,**e**) are 2 × 2 μm; surface height and Young’s modulus are indicated by color scales, next to the images.

We, next, tested the effectiveness of the approach, whereby metal nanoparticles would be encrusted into Retinin-based nanocoatings, prior to their self-assembly. We, further, argued that this approach could be more environmentally friendly, as metals would form bioconjugates and would be covered by the protein (Figure 4a). We chose silver and copper, as the metals for this approach.

Topology of the Retinin-based nanocoatings, formed in the presence of AgNO_3_, illustrates that Retinin maintains its self-assembly ability, under these conditions (Figure 4b). The resultant nano-structures have a characteristic size of 50–300 nm in diameter and up to 150 nm in height (Figure 4b,c), more pronounced than in the absence of metals (cf. Figure 3b,c).

The Young’s modulus of the Ag-metallized nanocoatings exceeded 30 GPa (Figure 4d), attaining values that are intermediate, between that of protein- and silver-covered surfaces [41,42,43], suggesting metallic reinforcement of the Retinin-based nanocoatings, in the manner that the metal nanoparticles become covered by the protein. The size of silver nanoparticles, in such a composite material, as estimated by the area of patches with higher Young’s modulus, does not exceed 100 nm—smaller than the characteristic size of the nano-structures (ca. 300 nm, Figure 4b), which, also, agrees with the idea that metal nanoparticles are hidden, within Retinin-based nano-structures.

**Figure 5:**
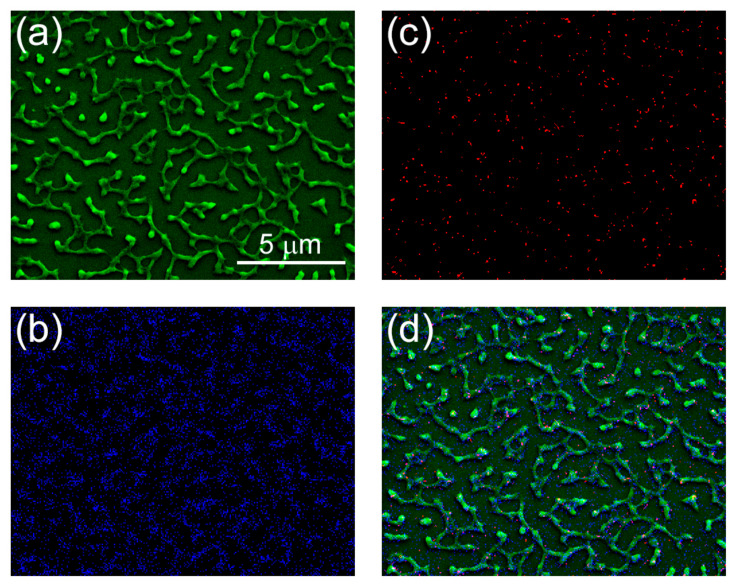
Scanning electron microscopy (**a**), combined with energy-dispersive X-ray spectroscopy, for carbon (**b**) and silver (**c**) of the Retinin-based nanocoatings, incorporating silver nanoparticles. (**d**) The merge of panels (**a**–**c**). Note, the overlap of carbon signals with the nanostructures, additionally, incorporates speckles of silver. Note, the bigger scale (15 × 10 μm) than in AFM images above.

In order to support these conclusions, we, additionally, performed scanning electron microscopy (SEM), followed by energy-dispersive X-ray spectroscopy (EDS), of the samples (Figure 5).

The structures observed using SEM differ from the images obtained using AFM (Figure 4b and Figure 5a, though note the different scales of the images). This difference could be due to the effect of plasma and vacuum on the protein-based nanostructures. Importantly, however, the elemental composition analysis confirmed colocalization of carbon and silver atoms in the nanostructures (Figure 5b–d), supporting our conclusions about the presence of silver nanoparticles, inside the nanocoatings.

We, further, found that CG13059, such as Retinin, can, also, incorporate silver in metal-bioconjugated nanocoatings (Figure 4e). Two other *Drosophila* Retinin-likes CG42718 and Nplp3 have similar properties, as does the Retinin-like CPR10 protein responsible for corneal nanocoating, in the mosquito A. gambae (Figure 4e). This versatility, of proteins capable of governing the Turing self-assembly on artificial surfaces, permits diversification of the functionalities of the resultant nanocoatings [12]. The section below provides comparative analysis of bactericidal, antiviral, and cytotoxic properties of such diverse nanocoatings, with different means of metal nanoparticle incorporation, through coalescence vs. bioconjugation.

### 3.4. Bactericidal, Antiviral, and Cytotoxic Properties of Diverse Composite Nanocoatings

**Figure 6:**
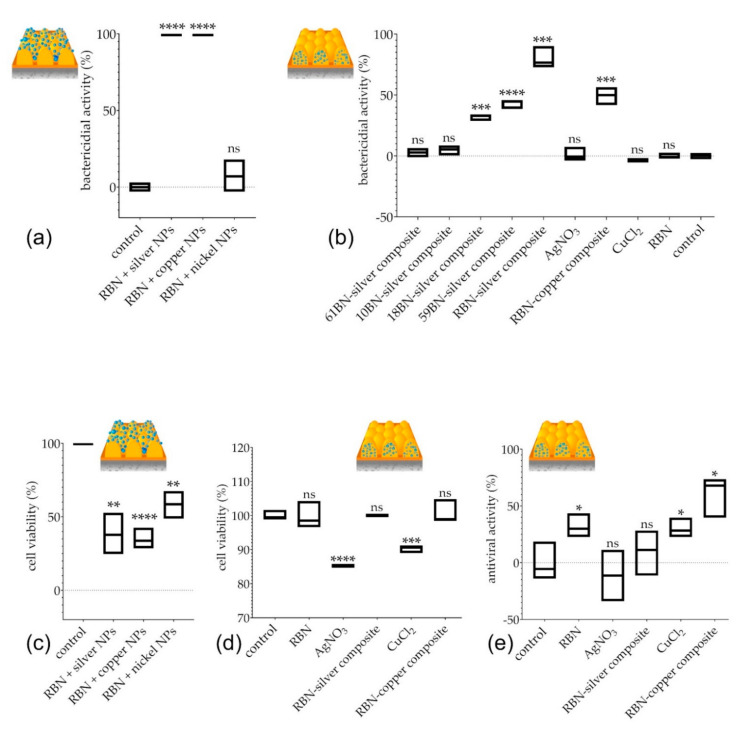
Bactericidal, antiviral, and cytotoxic properties of diverse metalized nanocoatings. (**a**) Metallization on top of pre-formed Retinin-based nanocoatings (RBN) completely eliminated bacteria, when containing silver or copper (but not nickel) nanoparticles (NPs). (**b**) Metallization, before formation of nanocoatings, provided high bactericidal activity, when Retinin-based nanocoatings (RBN) were built together with silver and copper composites. Silver composites with nanocoatings, based on Retinin-like proteins CG42718 and CG13059 (18BN-silver and 59BN-silver in the figure), were, also, bactericidal, unlike those based on Nplp3 (61BN-silver) or CPR10 (10BN-silver). Non-metalized RBN, as well as surface treatment with metallic salts alone, were ineffective. (**c**,**d**) Metallization on top of pre-formed Retinin-based nanocoatings (RBN) resulted in significant cytotoxicity (**c**), unlike metallization before formation of the nanocoatings (**d**). Note, different Y-axis scales in (**c**,**d**). (**e**) Antiviral properties of metalized bionic nanocoatings. Data are presented as mean ± SD, *n* = 3; “ns” indicates absence of significant difference, * indicates the *p*-value ≤ 0.05, ** indicates the *p*-value ≤ 0.01, *** *p*-value ≤ 0.001, **** *p*-value ≤ 0.0001.

We, first, analyzed bactericidal activities of surfaces with nickel, silver, or copper nanoparticles, formed on top of pre-formed Retinin-based nanocoatings, using NiSO_4_, AgNO_3_, and CuCl_2_. We found that the nanocoatings, encrusted with silver and copper but not nickel, can kill 100% of bacteria within 6 h (Figure 6a).

Next, we, similarly, assessed the bactericidal activities of metal-conjugated nanocoatings, self-assembled by Retinin or different Retinin-like proteins, finding significant bactericidal functionalities in silver-based composites of CG42718 and CG13059, but not of Nplp3 or *A. gambiae* CPR10 (Figure 6b). The highest bactericidal activity was identified in Retinin-silver nanoconjugates (Figure 6a,b); copper-based composites of Retinin were, also, effective.

Thus, among the five proteins analyzed, Retinin was superior in building bactericidal nanocoatings, upon armament with silver (and, to a lesser extent, copper) nanoparticles, provided either as bioconjugates integrated into the nanocoatings or as a layer above the nanocoatings. We note that, without metallization, Retinin-based nanocoatings did not possess bactericidal activities (Figure 6b).

To choose the safer method of metallization, we assessed the cytotoxic activity of the nanocoatings, against human embryonic kidney cells HEK-293 (Figure 6c,d). We suspected that metal nanoparticles formed on top of the Retinin-based nanocoatings potentially could have stronger cytotoxic adverse effects, through facilitated cellular uptake [37]. Indeed, we found a significantly (two-fold) reduced viability of HEK-293 cells, after exposure to the Retinin-based nanocoatings armed with silver, copper, or, even, nickel nanoparticles (Figure 6c). In contrast, no cytotoxicity was observed for the composite bionic nanocoatings containing Retinin and silver or copper (Figure 6d).

Inspired by the efficient bactericidal activity of the metal-Retinin composite nanocoatings, not charged with associated cytotoxicity, we, further, tested potential antiviral functionality of the nanocoatings. Interestingly, already, the non-metalized Retinin-based nanocoatings possessed a clear antiviral activity (Figure 6e). This activity was strongly enhanced by the copper conjugation but not by the silver conjugation (Figure 6e).

Thus, we conclude that metallic composites of Retinin-based bionic nanocoatings, while being safe for human cells, gain efficient bactericidal and antiviral functionalities; the former are more pronounced for the silver-nanoparticle-based composites, and the latter are more pronounced for the nanoparticle-based composites of copper.

## 4. Discussion

The COVID-19 pandemic has claimed many lives and challenged the effectiveness of many countries’s public health systems. It has, also, interfered with essential regular health services and surgical procedures. Other viral infections, evolving to cross the animal-to-human barrier, can, also, be anticipated [18]. Another future global health problem is expected to be the spread of antibiotic-resistant bacteria, feared by many to bring the effectiveness of our health systems back to the Middle Ages. The method we developed in this paper may reduce or even prevent the spread of both viruses and antibiotic-resistant bacteria.

The field of antimicrobial nanocoatings has been growing, exponentially. Interesting approaches, to achieve bactericidal functionalities, involve application of ZnO nanocomposites [44], TiO_2_ nanorods [45], and polyurethane micro/nanofibers ornamented with CuO nanocrystals [46]. These, and many other promising technologies being developed, however, are, mainly, applicable to relatively small areas of solid surfaces made of glass or stainless steel and, typically, require a complex application process [27,28,33]. In contrast, the method of functional nanocoating we propose in this work can be applied to varying (in area, texture, and materials) surfaces, including plastic.

Our technology is highly versatile, in the sense that Retinin, as the protein at the core of the nanoscale-self-assembly process, can be genetically or biochemically modified to adopt additional functionalities and improvements. Among such future modifications, we can consider (i) those speeding up the metal-nanoparticle-nucleation process [36], (ii) those adding bactericidal/antiviral-cationic peptides [47], (iii) those utilizing fatty acids (which are the other Turing component of the self-assembly mechanism) with additional anti-infective properties [48], and (iv) expansion of the list of metals and their oxides used in the functionalization, to include, e.g., Zn, ZnO, CuO, or TiO_2_ [45,49,50]. Such modifications can be expected to enhance the bactericidal and antiviral properties of the nanocoatings and to reduce possible adverse effects (Figure 1d).

In this work, we continue our journey, from understanding the building blocks and self-assembly mechanisms of bionanocoatings to translation of this knowledge towards technological applications [3,8,9,10,11,12,13,14,15]. The versatility of bionic nanocoatings that we can build using diverse Retinin-like proteins and waxes/fatty acids [11,12] has been multiplied in this study, by the diversity of metallic armaments. In the future, further means of derivatization of bionic nanocoatings can be conceivable, encompassing metallic layering or multi-layering (Figure 1e), as well as incorporation of other types of objects of the nanoscale (Figure 1f), for diverse applications in photonics, electronics, and medicine [51,52,53,54].

## Data Availability

The data presented in this study are fully available in the main text and Supplementary Materials of this article.

## Supplementary Materials

The following supporting information can be downloaded at: https://www.mdpi.com/article/10.3390/nano12111868/s1. Table S1: mass-spectrometry analysis of corneal proteins from *A. gambiae*. Supplementary Figure S1: alignment of amino acid sequences of Retinin of *D. melanogaster* and CPR10 of *A. gambiae*.
