# Supplementary material for: Bactericidal and Antiviral Bionic Metalized Nanocoatings
Source: Nanomaterials (Basel). 2022 May 30;12(11):1868. doi: 10.3390/nano12111868 (PMC9182136; doi:10.3390/nano12111868)
Supplement: Supplementary file 1 [file nanomaterials-12-01868-s001.zip › nanomaterials-1725494-supplementary.pdf]

nanomaterials

MDPI

Supplementary Materials

Bactericidal and Antiviral Bionic Metalized Nanocoatings

Mikhail Kryuchkov [1], Jozef Adamcik [2] and Vladimir L. Katanaev [1,3,*]

[1] Department of Cell Physiology and Metabolism, Translational Research Centre in Oncohaematology, Faculty of Medicine, University of Geneva, 1211, Geneva, Switzerland; mikhail.kryuchkov@unige.ch
[2] Adolphe Merkle Institute and National Center of Competence in Research Bio-Inspired Materials , University of Fribourg, 1700 Fribourg, Switzerland; jozef.adamcik@unifr.ch
[3] Institute of Life Sciences and Biomedicine, Far Eastern Federal University, 690922 Vladivostok, Russia
* Correspondence: vladimir.katanaev@unige.ch; Tel.: +41-22-379-5353

```
*
 BAD AVG GOOD
*
CPR10      :  50
Retinin    :  52
cons       :  50

CPR10      MASKISLIAVGLLLVNVGVNAQQYGQQLGRRSSQDRLNQLRSYDDGARQS---RNYNDLYNEQR--YASRTQ
Retinin    M-SRLFLPVLAIVLVSIGASHTAS---LEWPSNLVALSSVKSSQLLPIASEDSVELADGSSGSVSSSAAQPE

cons       * *:: * .:.::**.:*..       *   *.   *..::* :  .  *    :  *  . .    *::.:

CPR10      DQEQQQQQQHQDRRESSDYDRDDYSYGYAVRDELSGDIKS--QQEVRNGDRVRGQYRTLESDGTERIVDYTA
Retinin    DQSQEEAEEQQVSSASSG--SADPISGRLVSAGIPVSVPLPLILAARNGLRTVLTIQEPAVAKVGEVVQHVP

cons       **.*:: :::*    **.    *   *  *   :. .:       .*** *.    :      . .:*::..

CPR10      DDVRGFNAVVRHQPSVGTRAQLVHTLQPAVL-LRQPTVGHL-VSGQHRPALLTTPQQTSTVLLRN
Retinin    TAVSHQTQT------------VVHDHRRLVTPIVAPAVRTTQVIRQQPPLLWSVASDPRVVLIRN

cons         *   . .            :**  :  *  :  *:*    *  *: * * :...:. .**:**
```

Figure S1. Alignment of amino acid sequences of Retinin of *D. melanogaster* and CPR10 of *A. gambiae*, performed with the M-Coffee algorithm. Identical amino acids are marked with '*', similar – with ':', or '.' symbols. The total length of the proteins is 200 (CPR10) and 191 (Retinin) amino acids. Regions of good homology are colored in red. The main score is equal to 50 (a value of a 100 means full agreement between the considered alignment).

Table S1. Mass-spectrometry analysis of corneal proteins from *A. gambiae*.

Band size/Identified protein	Mv	Quantitative value (normalized total spectra)	Percentage of total spectra	Percentage of coverage	Exclusive unique peptide count	Exclusive unique spectrum count
Anopheles gambiae 13kDa AGAP006001-PA OS=Anopheles gambiae GN=CPR26 PE=4 SV=3	11 kDa	28	0.52%	71%	8	17
AGAP002994-PA OS=Anopheles gambiae GN=CPR10 PE=4 SV=3	23 kDa	23	0.42%	44%	11	20
Peptidyl-prolyl cis-trans isomerase OS=Anopheles gambiae GN=FKBP12 PE=4 SV=1	12 kDa	20	0.36%	80%	9	16
Cluster of AGAP010098-PA OS=Anopheles gambiae GN=CPR83 PE=4 SV=2 (Q7Q0W8_ANOGA)	17 kDa	18	0.34%	61%	7	12

AGAP003325-PA OS=Anopheles gambiae GN=AgaP_AGAP003325 PE=4 SV=1	9 kDa	18	0.34%	61%	8	13
AGAP000023-PA OS=Anopheles gambiae GN=AgaP_AGAP000023 PE=4 SV=3	42 kDa	17	0.32%	34%	11	15
AGAP006283-PB OS=Anopheles gambiae GN=CPR70 PE=4 SV=4	15 kDa	16	0.30%	57%	6	15
AGAP010122-PA OS=Anopheles gambiae GN=CPR132 PE=4 SV=1	44 kDa	14	0.26%	28%	8	12
AGAP007768-PA OS=Anopheles gambiae GN=AgaP_AGAP007768 PE=4 SV=1	9 kDa	14	0.26%	46%	8	12
Cluster of Profilin OS=Anopheles gambiae GN=AgaP_AGAP009861 PE=3 SV=3 (Q5TP19_ANOGA)	14 kDa	14	0.26%	79%	4	6
Anopheles gambiae 15kDa						
Cluster of AGAP010098-PA OS=Anopheles gambiae GN=CPR83 PE=4 SV=2 (Q7Q0W8_ANOGA)	17 kDa	37	0.96%	81%	16	16
AGAP002994-PA OS=Anopheles gambiae GN=CPR10 PE=4 SV=3	23 kDa	32	0.83%	63%	18	18
AGAP010957-PA (Fragment) OS=Anopheles gambiae GN=AgaP_AGAP010957 PE=4 SV=4	17 kDa	26	0.69%	82%	2	2
AGAP001274-PA OS=Anopheles gambiae GN=AgaP_AGAP001274 PE=3 SV=2	17 kDa	22	0.58%	59%	7	7
AGAP003375-PA OS=Anopheles gambiae GN=CPR114 PE=4 SV=4	17 kDa	20	0.52%	77%	10	10
Nucleoside diphosphate kinase OS=Anopheles gambiae GN=AgaP_AGAP007120 PE=3 SV=4	19 kDa	15	0.38%	71%	8	8
AGAP007082-PA OS=Anopheles gambiae GN=AgaP_AGAP007082 PE=4 SV=4	21 kDa	15	0.38%	67%	11	11
Eukaryotic translation initiation factor 5A OS=Anopheles gambiae GN=AgaP_AGAP011284 PE=3 SV=3	18 kDa	14	0.37%	81%	12	12
AGAP007841-PA (Fragment) OS=Anopheles gambiae GN=AgaP_AGAP007841 PE=3 SV=4	18 kDa	14	0.37%	70%	10	10
AGAP010122-PA OS=Anopheles gambiae GN=CPR132 PE=4 SV=1	44 kDa	13	0.35%	37%	11	11
Anopheles gambiae 20kDa						
AGAP002994-PA OS=Anopheles gambiae GN=CPR10 PE=4 SV=3	23 kDa	155	2.00%	82%	28	51
AGAP003599-PA OS=Anopheles gambiae GN=AgaP_AGAP003599 PE=3 SV=3	22 kDa	36	0.47%	51%	11	23

Cluster of AGAP006829-PA OS=Anopheles gambiae GN=CPR59 PE=4 SV=4 (Q7QIG4_ANOGA)	22 kDa	36	0.47%	79%	12	21
AGAP010122-PA OS=Anopheles gambiae GN=CPR132 PE=4 SV=1	44 kDa	33	0.44%	36%	10	22
AGAP007963-PA OS=Anopheles gambiae GN=AgaP_AGAP007963 PE=4 SV=3	22 kDa	32	0.42%	59%	12	21
AGAP011131-PA OS=Anopheles gambiae GN=AgaP_AGAP011131 PE=4 SV=3	20 kDa	31	0.40%	70%	11	20
Cluster of Glutathione S-transferase 1, isoform D OS=Anopheles gambiae GN=GstD1 PE=1 SV=1 (GST1D_ANOGA)	23 kDa	31	0.40%	42%	10	20
AGAP000970-PA OS=Anopheles gambiae GN=AgaP_AGAP000970 PE=4 SV=1	20 kDa	28	0.37%	78%	12	21
40S ribosomal protein S7 OS=Anopheles gambiae GN=RpS7 PE=2 SV=2	22 kDa	27	0.35%	65%	12	18
AGAP011054-PA OS=Anopheles gambiae GN=TPX2 PE=4 SV=2	22 kDa	27	0.35%	48%	11	19
Anopheles gambiae 23kDa						
AGAP002994-PA OS=Anopheles gambiae GN=CPR10 PE=4 SV=3	23 kDa	230	3.40%	82%	32	64
Cluster of AGAP006829-PA OS=Anopheles gambiae GN=CPR59 PE=4 SV=4 (Q7QIG4_ANOGA)	22 kDa	40	0.58%	83%	12	24
Citrate synthase OS=Anopheles gambiae GN=AgaP_AGAP012048 PE=3 SV=4	52 kDa	34	0.50%	43%	19	28
Cluster of AGAP004146-PA OS=Anopheles gambiae GN=AgaP_AGAP004146 PE=3 SV=4 (Q7QB74_ANOGA)	22 kDa	33	0.48%	57%	19	26
Triosephosphate isomerase OS=Anopheles gambiae GN=AgaP_AGAP001630 PE=3 SV=4	26 kDa	32	0.47%	82%	17	20
Cluster of Actin-5C OS=Anopheles gambiae GN=Act5C PE=2 SV=1 (ACT5C_ANOGA)	42 kDa	24	0.35%	30%	12	21
AGAP011824-PA OS=Anopheles gambiae GN=TPX4 PE=4 SV=3	25 kDa	24	0.35%	71%	16	20
Cluster of AGAP006146-PA OS=Anopheles gambiae GN=CPLCA2 PE=4 SV=4 (Q7Q5X8_ANOGA)	20 kDa	23	0.33%	74%	12	16
Cytochrome b-c1 complex subunit Rieske, mitochondrial OS=Anopheles gambiae GN=AgaP_AGAP008955 PE=4 SV=4	28 kDa	23	0.33%	43%	9	20
AGAP005061-PB OS=Anopheles gambiae GN=AgaP_AGAP005061 PE=3 SV=2	23 kDa	23	0.33%	43%	14	16
Anopheles gambiae 36kDa						

Cluster of AGAP001799-PB OS=Anopheles gambiae GN=AgaP_AGAP001799 PE=3 SV=1 (F5HME4_ANOGA)	32 kDa	41	0.75%	65%	25	42
AGAP010122-PA OS=Anopheles gambiae GN=CPR132 PE=4 SV=1	44 kDa	38	0.68%	40%	13	26
Glycerol-3-phosphate dehydrogenase [NAD(+)] OS=Anopheles gambiae GN=AgaP_AGAP007593 PE=3 SV=1	39 kDa	35	0.63%	67%	21	37
Cluster of Annexin OS=Anopheles gambiae GN=ANXB10C PE=3 SV=4 (Q7PS96_ANOGA)	36 kDa	30	0.55%	61%	18	28
Cluster of AGAP011050-PA OS=Anopheles gambiae GN=AgaP_AGAP011050 PE=4 SV=3 (Q7PF06_ANOGA)	36 kDa	30	0.55%	77%	11	26
Cluster of AGAP010929-PA OS=Anopheles gambiae GN=AgaP_AGAP010929 PE=3 SV=4 (Q7PSI4_ANOGA)	50 kDa	29	0.53%	49%	20	18
Cluster of Actin-5C OS=Anopheles gambiae GN=Act5C PE=2 SV=1 (ACT5C_ANOGA)	42 kDa	27	0.48%	56%	17	28
Glyceraldehyde-3-phosphate dehydrogenase OS=Anopheles gambiae GN=AgaP_AGAP009623 PE=3 SV=2	35 kDa	27	0.48%	67%	16	28
AGAP000094-PA OS=Anopheles gambiae GN=AgaP_AGAP000094 PE=3 SV=5	35 kDa	27	0.48%	71%	16	27
AGAP001506-PA OS=Anopheles gambiae GN=AgaP_AGAP001506 PE=4 SV=4	38 kDa	25	0.45%	59%	14	26
Anopheles gambiae 38kDa						
Cluster of AGAP005627-PC OS=Anopheles gambiae GN=AgaP_AGAP005627 PE=3 SV=1 (A7UTS9_ANOGA)	40 kDa	69	1.20%	61%	25	50
Cluster of Fructose-bisphosphate aldolase OS=Anopheles gambiae GN=AgaP_AGAP002564 PE=3 SV=1 (F5HKV6_ANOGA)	39 kDa	51	0.92%	74%	20	45
Cluster of AGAP005079-PD OS=Anopheles gambiae GN=AgaP_AGAP005079 PE=4 SV=1 (A7UT59_ANOGA)	42 kDa	42	0.75%	64%	22	37
AGAP011172-PA OS=Anopheles gambiae GN=AgaP_AGAP011172 PE=3 SV=3	43 kDa	35	0.64%	83%	19	35
Isocitrate dehydrogenase [NAD] subunit, mitochondrial OS=Anopheles gambiae GN=AgaP_AGAP002728 PE=3 SV=1	45 kDa	34	0.62%	46%	16	29

AGAP010122-PA OS=Anopheles gambiae GN=CPR132 PE=4 SV=1	44 kDa	32	0.59%	37%	11	23
AGAP009783-PA (Fragment) OS=Anopheles gambiae GN=AgaP_AGAP009783 PE=3 SV=3	46 kDa	32	0.59%	57%	18	33
Malate dehydrogenase (Fragment) OS=Anopheles gambiae GN=AgaP_AGAP009510 PE=3 SV=3	34 kDa	32	0.59%	61%	24	31
Cluster of Actin-5C OS=Anopheles gambiae GN=Act5C PE=2 SV=1 (ACT5C_ANOGA)	42 kDa	31	0.55%	60%	19	32
Aspartate aminotransferase OS=Anopheles gambiae GN=AgaP_AGAP009685 PE=3 SV=3	47 kDa	31	0.55%	54%	18	31
Anopheles gambiae 52kDa AGAP010122-PA OS=Anopheles gambiae GN=CPR132 PE=4 SV=1	44 kDa	85	1.50%	44%	17	32
Cluster of AGAP010929-PA OS=Anopheles gambiae GN=AgaP_AGAP010929 PE=3 SV=4 (Q7PSI4_ANOGA)	50 kDa	51	0.93%	72%	17	33
ATP synthase subunit alpha OS=Anopheles gambiae GN=AgaP_AGAP005134 PE=3 SV=2	59 kDa	40	0.72%	53%	25	37
Cluster of AGAP001218-PA OS=Anopheles gambiae GN=AgaP_AGAP001218 PE=3 SV=5 (Q7PUE2_ANOGA)	50 kDa	36	0.66%	60%	21	36
AGAP000901-PA OS=Anopheles gambiae GN=AgaP_AGAP000901 PE=4 SV=5	61 kDa	34	0.62%	56%	26	13
AGAP005293-PB OS=Anopheles gambiae GN=AgaP_AGAP005293 PE=3 SV=1	51 kDa	30	0.54%	45%	9	34
Arrestin 2-like protein Arr2 OS=Anopheles gambiae str. PEST GN=ARR2 PE=4 SV=1	45 kDa	29	0.53%	61%	23	32
AGAP010134-PA OS=Anopheles gambiae GN=Arr1 PE=2 SV=1	43 kDa	27	0.50%	61%	23	27
AGAP005558-PA OS=Anopheles gambiae GN=AgaP_AGAP005558 PE=3 SV=1	52 kDa	27	0.50%	52%	18	32
AGAP002306-PA OS=Anopheles gambiae GN=AgaP_AGAP002306 PE=4 SV=4	49 kDa	27	0.50%	42%	21	27
